# Death from Inhalation of Ether

**Published:** 1867-10

**Authors:** 


### SELECTED ARTICLES.

### ARTICLE V.

### *Death from Inhalation of Ether.*

HITHERTO it has been the boast of the advocates of ether that not a single well attested case is on record, of death resulting immediately from its inhalation. Such a case is reported in the Paris *Gazette Hebdomadaire* for May, communicated to the Medical Society of Lyons by M. Laroyenne, in whose hands it occurred. The subject appears to have been a cripple in feeble health. The nature of the operation performed, does not fully appear. We translate the account as its stands, together with the comments, that the reader may attach his own estimate to it.—"Subject, a female aged 48 years, constitution feeble. Has an old affection of the left knee, with distortion of the lower extremities. Anæsthesia practiced with caution, insensibility occurring after the inhalation of ten drachms of ether. In two or three minutes, the breathing became embarrassed, face pale, pulse insensible. The recumbent position and cold affusions roused her from this first syncope. Hardly had she been put in bed—fifteen minutes after the first syncope—when a new attack came on, and notwithstanding artificial respiration, prolonged insufflation, galvanization of the diaphragm and the intercostals, galvanization of the heart by the aid of long acupuncture needles, it was impossible to recall her to life. An attentive examination of the thoracic organs before anesthesia, had failed to demonstrate any organic lesion. The analysis of the ether by Professor Glenard proved that it contained no foreign substance other than three parts in a hundred of water. *Necropsy.* Mucus in the larynx and trachea, pleural adhesions ; tubercles in the left pleura ; base of left lung congested. The pulmonary tissue was impregnated with the odor of ether. Small quantity of fluid in the pericardium ; heart normal ; ventricles empty ; auricles gorged with blood ; nervous centers sound, possessing a feeble odor of ether ; a small quantity of fluid in the ventricles. Medulla compressed by a tubercular mass developed in the seventh and eighth dorsal vertebræ, which presented no appearances from without. The alteration had advanced to the left coxo-femoral articulation, which presented tubercular masses and osseous fragments of the capsule ; head of the femur completely detached ; neck totally destroyed. Right hip joint full of blood ; recent fracture of the neck of the femur. This case should be considered as an example of death consecutive to etherization. Among analogous cases in which death has supervened some time after etherization, it has been possible, too often, to raise serious doubts on the nature of the accidents. Here the time which elapsed between anæsthesia and death was too short for the influence of the ether to be discarded. M. Laroyenne has himself passed judgment after the following manner : "Very evidently the ether was not the sole cause of death in the present case. It acted in favoring the syncope to which the patient was already pre-disposed by reason of her state of general debility. Assuredly the syncope would not have been produced had the operation been effected without etherization."

In a subsequent number of the same journal, from which the above is extracted, appears a short account of an interesting discussion in the Medical Society of Lyons, called up by a report of this death from ether. One of the members stated that during the past fourteen years there had been *seven* deaths attributed to ether—this, notwithstanding the former announcement of M. Petrequin, that since chloroform had been discarded and ether substituted some fourteen years ago in Lyons, no deaths following anæsthesia had occurred in the hospitals of that city. It may be added, however, that the seven cases were submitted to the criticism of the members of the Society, and it was asserted that in almost all of them, the ether had less to do in causing death, than the unfortunate complications existing in the patients. And we may add, that this is in accordance with the received opinion, at least, in America, that death does not occur *immediately* as the consequent of the ether, but *remotely*, and only in those cases where serious complications exist, and which in many instances should contra-indicate its use. The Society adopted a resolution that hereafter all deaths chargeable to ether should be submitted to it, by a Commission, for discussion.—*Pacific Medical and Surgical Journal.*

